# Detection of Herpes Simplex Virus Type-1 in Patients with Fibrotic Lung Diseases

**DOI:** 10.1371/journal.pone.0027800

**Published:** 2011-12-20

**Authors:** Ismini Lasithiotaki, Katerina M. Antoniou, Virginia-Maria Vlahava, Konstantinos Karagiannis, Demetrios A. Spandidos, Nikolaos M. Siafakas, George Sourvinos

**Affiliations:** 1 Laboratory of Molecular and Cellular Pulmonology, Medical School, University of Crete, Heraklion, Crete, Greece; 2 Laboratory of Virology, Medical School, University of Crete, Heraklion, Crete, Greece; 3 Department of Thoracic Medicine, Medical School, University of Crete, Heraklion, Crete, Greece; Naval Research Laboratory, United States of America

## Abstract

The current study intends to investigate i) the incidence of herpes viruses including Herpes Simplex Virus type-1 (HSV-1), Cytomegalovirus (CMV) and Human Herpes Virus -6, -7, -8 (HHV6, HHV7, HHV8) in two biological samples, bronchoalveolar lavage fluid (BALF) and lung tissue biopsy, in different forms of pulmonary fibrosis, and ii) the induction of molecular pathways involved in fibrosis by herpesvirus infection in primary cell cultures. PCR was employed for the detection of CMV, HHV6-8 and HSV-1 DNA in lung specimens (4 controls and 11 IPF specimens) and BALF pellet [6 controls and 20 fibrotic Idiopathic Intestitial Pneumonias (f-IIPs) samples: 13 idiopathic pulmonary fibrosis (IPF) and 7 nonspecific idiopathic interstitial pneumonia (NSIP)] samples. Among all herpesviruses tested, HSV-1 was detected in 1/11 (9%) specimens from IPF lung tissue and in 2/20 (10%) samples of f-IIPs BALF whereas the control group was negative. Primary cell cultures from BALF of patients with IPF and healthy controls were infected *in vitro* with wild-type HSV-1 virus and Real Time PCR was employed for the detection of gene transcription of specific axes implicated in lung fibrosis. Primary cell cultures were permissive to HSV-1, resulting in an upregulation of the fibrotic growth factors TGFβ1 and FGF, the angiogenetic markers SDF1a, SDF1b, VEGF, FGF and the regulators of tissue wound healing MMP9 and CCR7. Downregulation was noted for the CXCR4 and MMP2 genes, while a different response has been detected in healthy donors regarding the expression of the aforementioned markers. These results implicate for the first time the HSV-1 with Fibrotic Idiopathic Interstitial Pneumonias since the virus presented similar incidence in two different biological samples.

## Introduction

Idiopathic pulmonary fibrosis (IPF) is perhaps the most pernicious and enigmatic form of a greater problem of lung fibrogenesis with no proven effective therapy other than lung transplantation and associated with a median patient survival of three years [1]. The pathogenesis of IPF remains unknown. Epithelial injury, fibroblast activation and repetitive cycles of injury and abnormal repair are almost certainly key events [2]. While the pathogenesis remains unclear, the spatial and temporal variance of fibrotic lesions suggests a repeated stimulus causing lung injury over the course of the disease [3].

Several studies have implicated chronic viral infection as a cause of ongoing epithelial injury in IPF and therefore an important cofactor, either initiating or exacerbating the disease [4]. Previous studies have shown the presence of Epstein-Barr virus (EBV), Cytomegalovirus, and human herpesviruses (HHV) 7 and 8 in patients with IPF [5]–[7]. It should be noted that other studies did not find an association between herpesviral infection and IPF, and it is not clear whether these discrepancies represent geographical variation or technical differences [8], [9].

Animal models support the hypothesis linking herpesvirus infections with specific T helper 2 (Th2) cytokine profile and alternative activation of macrophages as co-factors for the development of pulmonary fibrosis [4], [10]–[12]. The identified mechanisms contributing to lytic virus-induced exacerbation of fibrosis include Th2 bias, persistent reactivation, alternative activation of macrophages, and fibrocyte recruitment [3], [12]–[15]. Moreover, novel data indicate that herpesvirus promotes epithelial-mesenchymal transition, a crucial process which occurs during fibrosis [3], [16].

Finally, recent research suggests potential gammaherpesvirus-mediated mechanisms for the increased fibrosis involving alveolar epithelial cells (AECs) and macrophages [17]. Latently infected AECs also produce higher levels of cysteinyl leukotrienes and the potent pro-fibrotic cytokine transforming growth factor (TGF)-β1 through alteration of Cux1/Wnt signalling [17], [18].

This study aimed to investigate the incidence of a wide range of herpesviruses, including HSV-1, CMV and the most recently discovered HHV6, HHV7 and HHV8, in two different biological samples, bronchoalveolar lavage fluid (BALF) and lung tissue, in different forms of pulmonary fibrosis. The hypothesis tested was that a herpes virus infection, through induction of molecular pathways, could promote the fibrotic pathway. The results support the hypothesis only for the HSV-1.

## Results

### Incidence of Herpesviruses in BALF and lung tissue

**Figure 1:**
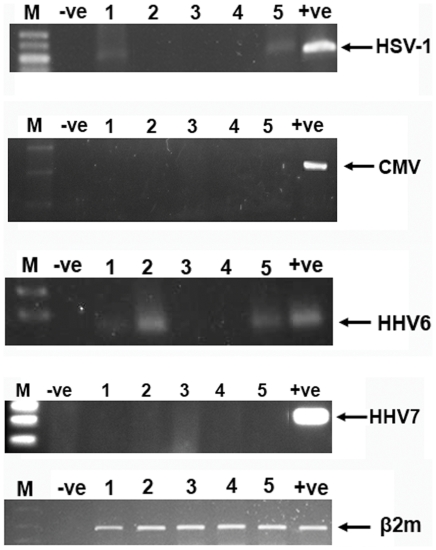
Detection of Herpesvirus genomes in BALF and lung tissue. M; molecular weight marker, +ve; positive control, −ve; negative control; β2m: beta-2-microglobulin.

All lung tissue biopsy samples and all BALF specimens (patients and controls) were negative for the presence of CMV, HHV7 and HHV8 DNA. HSV-1 was detected in 1 out of 11 (9%) specimens from the IPF lung tissue group while 2 out of 20 (10%) samples of fibrotic Idiopathic Intestitial Pneumonias (f-IIPs) BALF were also positive for the same virus. Lung tissues biopsies or BALF control specimens proved to be PCR-negative for HSV-1. HHV6 DNA was also not detected in lung tissue biopsies of patients and controls. However, 3 out of 6 (3/6, 50%) of the controls and 9 out of 20 (45%) f-IIPs patients BALF were found positive for the same virus. Representative examples of herpesvirus-positive samples are shown in Figure 1 while the cumulative results are presented in Table S1.

### Primary cell culture

**Figure 2:**
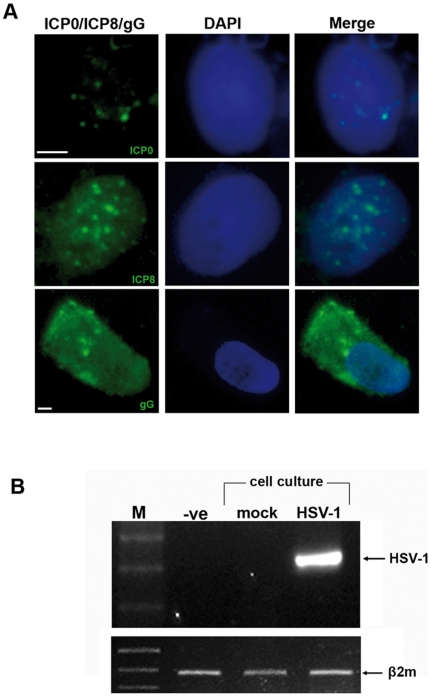
A. Expression of ICP0 (immediate early), ICP8 (early) and gG (late) HSV-1 proteins in infected macrophages. DAPI (4,6-diamidino-2-phenylindole). Magnification, ×600 (bars: 5 µM). B. Confirmation of successful HSV-1 infection of cultured lung macrophages by PCR.

The permissiveness of the primary cultures by HSV-1 was evaluated by immunofluorescence. After *in vitro* infection with a wild-type HSV-1 strain, macrophages expressed all classes of HSV-1 genes such as ICP0 (immediate early), ICP8 (early) and gG (late) genes, during the course of the infection, as observed by fluorescence microscopy (Figure 2A). In addition, the HSV-1 infected primary cell cultures tested positive for the genomic DNA of the virus, using PCR methodology, while the mock infected primary cell cultures tested negative for the presence of viral genome (Figure 2B). These results demonstrate that pulmonary macrophages are permissive to the virus and can serve as hosts in lung tissue.

### Gene expression

We next sought to address whether infection of macrophages by HSV-1 might cause alterations in the expression pattern of fibrotic growth factors [Transforming Growth Factor β1 (TGFβ1) and Fibroblast Growth Factor (FGF)], angiogenetic markers [Stromal cell-Derived Factor 1a and 1b (SDF-1a, SDF-1b), C-X-C chemokine receptor type 4 (CXCR4), Vascular Endothelial Growth Factor (VEGF)], innate immunity factors [Toll-Like Receptor 3, 8, 9 (TLR3, TLR8, TLR9)] and regulators of tissue wound healing [Matrix Metallopeptidase 2, 9 (MMP2, MMP9) and C-C chemokine receptor type 7 (CCR7)] with ligands Chemokine (C-C motif) ligand 21 and 19 (CCL21, CCL19). Primary cell cultures from fresh BALF were first tested negative for the presence of HSV-1 DNA and were subsequently divided into two samples, only one of which was *in vitro* infected with wild-type HSV-1. The potential alterations in the expression level of various genetic axes were assessed by real-time PCR, revealing intriguing differences among the infected and non-infected cell.

### Fibrotic and angiopoietic axis

**Figure 3:**
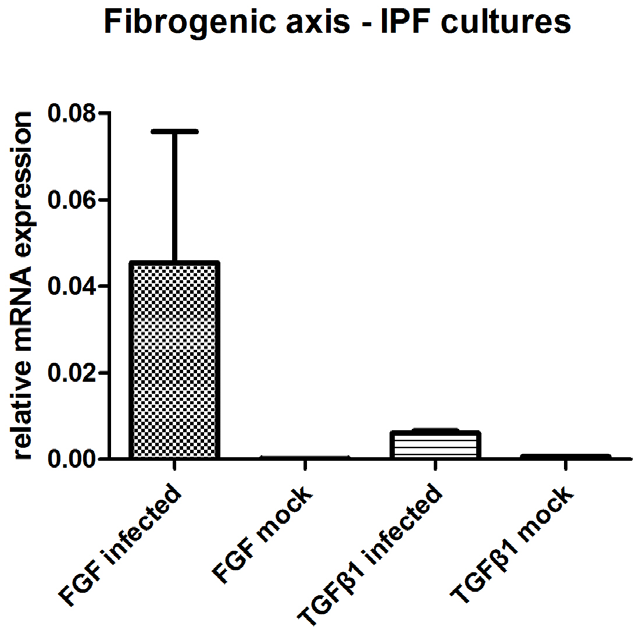
mRNA expression profile of fibrogenic axis in HSV-1 (n = 4) and mock infected (n = 4) macrophages from IPF patients.

**Figure 4:**
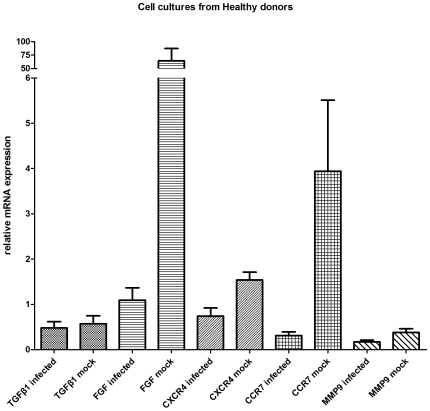
mRNA expression profile of HSV-1 (n = 3) and mock infected (n = 3) macrophages from healthy donors.

The fibrotic axis was upregulated in the HSV-1 infected cells of IPF patients in comparison to the mock cultures, with higher transcript levels of TGFβ1 (mean±SD, 0.0057±0.001 versus 0.0006±0.0001) and FGF (mean±SD, 0.033±0.066 versus 0.0001±0.0001) (Table 1). The ratio of the means for the two culture groups was 9.5 and 330, respectively, favoring the infected cultures (Figure 3). On the contrary, the fibrogenic axis in the primary cell cultures of healthy donors was downregulated in the HSV-1 infected cells compared to the mock cultures with lower transcript leves of TGFβ1 (mean±SD, 0.48±0.24 versus 0.57±0.31) and FGF (mean±SD, 1.06±0.52 versus 64.36±39.48) and ratios of the means for the two culture groups 0.84 and 0.02, respectively for the two genes (Table 2), favoring the mock cultures (Figure 4).

**Table 1:** Expression profile of fibrogenic gene mRNA in infected with recombinant HSV-1 cultures and mock infected cultures from patients with IPF.

MEANS±SD	Infected(n = 4)	Mock(n = 4)	Ratio of means (infected/mock)	*P* value
SDF-1a	11.97±18.44	1.56±2.49	7.7	0.358
SDF-1b	0.09±0.019	0.00±0.0	-	0.390
CXCR4	0.0007±0.001	3.6±7.2	0.0002	0.391
VEGF	0.04±0.058	0.00±0.00001	-	0.263
TGF-β1	0.006±0.001	0.0006±0.0001	9.5	0.392
FGF	0.033±0.07	0.0001±0.0001	330	0.391
MMP2	0.003±0.006	0.004±0.007	0.84	0.391
MMP9	88.13±135.99	12.95±16.47	6.81	0.384
CCR7	6.61±13.19	0.3±0.55	22.3	0.392
CCL19	0.002±0.004	0.0005±0.0007	4	0.447
CCL21	0.0005±0.0008	0.0002±0.0002	2.5	0.513
TLR3	0.7±1.08	0.3±0.59	2.34	0.619
TLR8	0.0±0.0	2.68±4.22	0	0.294
TLR9	0.0±0.0	0.0±0.0	-	-

**Table 2:** Expression profile of fibrogenic gene mRNA in infected with recombinant HSV-1 cultures and mock infected cultures from healthy donors.

MEANS±SD	Infected(n = 4)	Mock(n = 4)	Ratio of means (infected/mock)	*P* value
SDF-1a	0.69±0.67	1.00±1.00	0.67	0.795
SDF-1b	1.12±1.12	0.9±0.16	1.23	0.864
CXCR4	0.74±0.31	1.57±0.25	0.47	0.101
VEGF	1.06±0.52	64.36±39.48	0.02	0.184
TGF-β1	0.48±0.24	0.57±0.31	0.84	0.828
FGF	1.06±0.52	64.36±39.48	0.02	0.184
MMP2	0.39±0.26	0.41±0.19	0.96	0.961
MMP9	0.17±0.07	0.38±0.14	0.45	0.265
CCR7	0.31±0.14	3.94±2.72	0.08	0.254
CCL19	17.83±8.92	2.09±0.87	8.53	0.154
CCL21	27000±2645.75	15000±2645.75	1.8	0.033
TLR3	33.33±33.33	21.33±8.59	1.56	0.745
TLR8	0.5±0.16	0.23±0.28	0.95	0.939
TLR9	5.06±4.29	49.31±24.46	0.10	0.149

**Figure 5:**
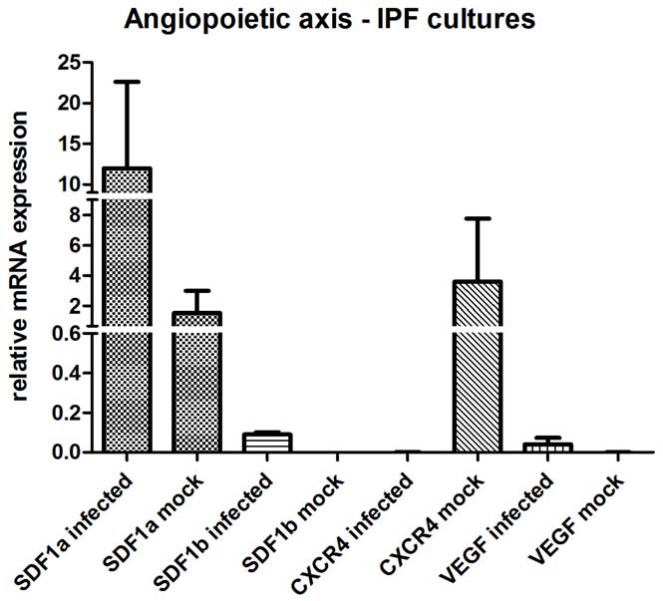
mRNA expression levels of angiopoietic axis in HSV-1 (n = 4) and mock infected (n = 4) macrophages from IPF patients.

The mRNA levels for SDF-1a, SDF-1b and VEGF of the angiopoietic axis were increased in the infected cultures of IPF patients as compared to the mock infected cultures. In particular, SDF-1a was expressed at higher levels in the infected cultures (mean±SD, 11.97±18.443 versus 1.56±2.49 for the mock infected cultures), with a ratio of means 7.7. In addition, the VEGF (mean±SD, 0.04±0.058 versus 0.0±0.0) and SDF-1b (mean±SD, 0.09±0.0188 versus 0.0±0.0) transcript levels were higher in the infected samples of IPF patients, compared to the mock infected cultures (Figure 5). Decreased expression levels were observed for the CXCR4 gene in the infected cultures (0.0007±0.00138), compared to the mock infected cultures of the same group of patients (mean±SD, 0.0007±0.001 versus 1.56±2.49), with a ratio of 0.0002 (Figure 5).

The mRNA levels of the genes involved in the angiopoietic axis were mainly decreased in the infected cultures of healthy donors compared to the mock infected cultures. Specifically, SDF-1a was expressed at lower levels in the infected cultures (mean±SD, 0.69±0.67 versus 1.0±1.0 for the mock infected cultures), with a ratio of means 0.67. In addition, VEGF (mean±SD, 1.06±0.52 versus 64.36±39.48) and CXCR4 (mean±SD, 0.74±0.31 versus 1.57±0.25) transcript levels were lower in the infected samples compared to the mock infected samples, with ratios of means 0.02 and 0.47 respectively (Figure 4). In contrast, increased expression levels were observed for the SDF-1b gene in the infected cultures compared to the mock infected cultures (mean±SD, 1.12±1.12 versus 0.9±0.16), with a ratio of 1.23.

The HSV-1 infected primary cultures from IPF patients showed lower expression levels in both axis compared to the infected primary cultures from healthy donors, with the only exception of SDF-1a (Table 3). Specifically, mRNA transcript levels for SDF-1b were lower in the cultures from IPF patients compared to the infected primary cultures from healthy donors with ratio of the means 0.08 (p = 0.348), as well as for CXCR4 with ratio of the means 0.001 (p = 0.568) and VEGF with ratio of the means 0.04 (p = 0.05). For the fibrogenic axes genes, the mRNA transcript levels for TGF-β1 were lower in the cultures from IPF patients compared to the infected primary cultures from healthy donors with ratio of the means 0.01 (p = 0.439), as well as for FGF with ratio of the means 0.03 (p = 0.463).

**Table 3:** Expression profile of mRNA transcript levels of axis in infected with recombinant HSV-1 from IPF patients compared to healthy donors.

*Infected with recombinant HSV-1 cultures*				
*Gene*	*IPF (MEANS)*	*Healthy Donors (MEANS)*	*Ratio of the means (IPF/Donors)*	*p value*
SDF-1a	11.97	0.69	17.35	0.348
SDF-1b	0.09	1.12	0.08	0.568
CXCR4	0.00	0.74	0.001	0.349
VEGF	0.04	1.06	0.04	0.05
TGF-β1	0.01	0.48	0.01	0.439
FGF	0.03	1.06	0.03	0.463
MMP2	0.00	0.39	0.01	0.132
MMP9	88.13	0.17	518.41	0.324
CCR7	6.61	0.31	21.32	0.7
CCL19	0.00	17.83	0.0001	0.246
CCL21	0.00	27000	0.00	0.001
TLR3	0.70	33.33	0.02	0.980
TLR8	0.00	0.5	0.00	0.014
TLR9	0.00	5.06	0.00	0.05

The mock infected primary cultures from IPF patients showed lower expression levels the fibrogenic axes compared to the mock infected primary cultures from healthy donors (Table 4). Specifically, mRNA transcript levels for TGF-β1 were lower in the cultures from IPF patients compared to the ones from healthy donors with ratio of the means 0.001 (p = 0.240), as well as for FGF with ratio of the means 0.000001 (p = 0.0.501). For the angiopoietic axes genes, the mRNA transcript levels for SDF-1b and VEGF were undetectable in the cultures from IPF patients while for the mock infected primary cultures from healthy donors were quantifiable with ratio of the means 0.00 (p = 0.01 and p = 0.076 respectively). For the SDF-1a and CXCR4 genes, the transcript levels were higher in the mock infected cultures from IPF patients, with ratios of the means 1.56 (p = 0.758) and 2.29 (p = 0.372), respectively compared to the mock infected from healthy donors.

**Table 4:** Expression profile of mRNA transcript levels of axis in mock infected with recombinant HSV-1 cultures from IPF patients compared to healthy donors.

*Mock infected cultures*				
*Gene*	*IPF (MEANS)*	*Healthy Donors (MEANS)*	*Ratio of the means (IPF/Donors)*	*p value*
SDF-1a	1.56	1	1.56	0.758
SDF-1b	0	0.9	0.00	0.01
CXCR4	3.6	1.57	2.29	0.372
VEGF	0	64.36	0.00	0.076
TGF-β1	0.0006	0.57	0.00	0.240
FGF	0.0001	64.36	0.00	0.501
MMP2	0.004	0.41	0.01	0.051
MMP9	12.95	0.38	34.08	0.254
CCR7	0.3	3.94	0.08	0.074
CCL19	0.0005	2.09	0.00	0.037
CCL21	0.0002	15000	0.00	0.001
TLR3	0.3	21.33	0.01	0.028
TLR8	2.68	0.23	11.65	0.431
TLR9	0	49.31	0.00	0.019

### Regulators of wound healing

**Figure 6:**
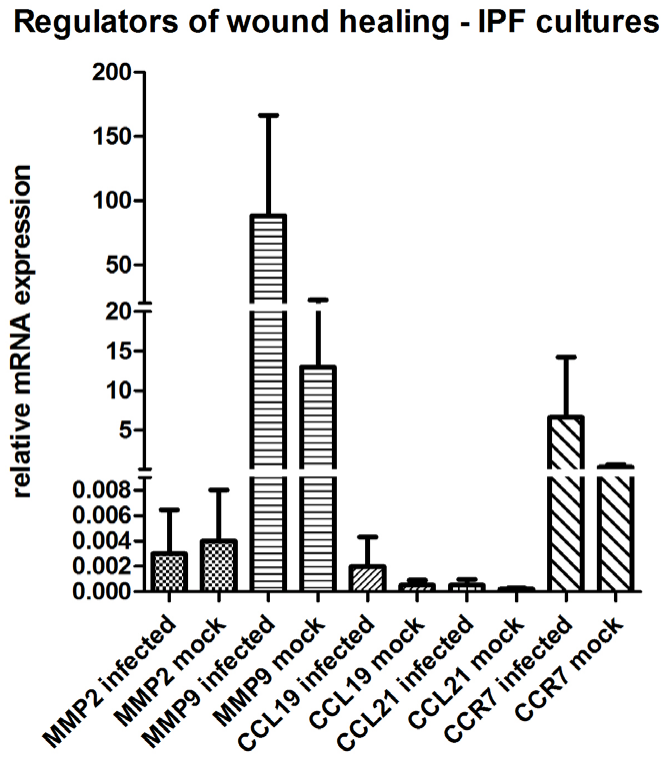
mRNA expression of regulators of wound healing in HSV-1 (n = 4) and mock infected (n = 4) macrophages from IPF patients.

Molecular analysis demonstrated elevated transcription levels for the regulators of wound healing in the HSV-1 infected cultures of patients with IPF, with the only exception of MMP2. More specifically, MMP9 transcript levels for the infected samples were higher compared to the mock (mean±SD, 88.13±135.99 versus 12.95±16.47) with a ratio of 6.81. CCR7 transcript levels were also higher for the infected samples (mean±SD, 6.61±13.19 versus 0.3±0.55) with a ratio of 22.31 (Figure 6). The mRNA levels of MMP2 were lower in the infected samples compared to the mock infected samples (mean±SD, 0.003±0.006 versus 0.004±0.007) with a ratio of 0.84.

In contrast, infected cultures from healthy donors showed lower transcription levels for the regulators of wound healing. MMP2 and MMP9 trancript levels for the infected samples were lower compared to the mock infected samples (mean±SD, 0.39±0.26 versus 0.41±0.19 and 0.17±0.07 versus 0.38±0.14, respectively) with ratios of means 0.96 and 0.45 respectively. CCR7 transcript levels were also lower for the infected samples (mean±SD, 0.31±0.14 versus 3.94±2.72) with a ratio of 0.08 (Figure 4).

The ligands of CCR7, CCL19 (mean±SD, 0.002±0.004 versus 0.0005±0.0007) and CCL21 (mean±SD, 0.0005±0.0008 versus 0.0002±0.0002) were overexpressed in the infected samples from IPF patients with a ratio of 4 and 2.5, respectively, over the expression of the mock infected samples (Figure 6). This was also the case for the infected cell cultures from healthy donors, with higher transcript levels of CCL19 (mean±SD, 17.83±8.92 versus 2.09±0.87) and CCL21 (mean±SD, 27000±2645.75 versus 15000±2645.75) compared to the mock infected cultures, with ratios of means 8.53 and 1.8 respectively.

The HSV-1 infected primary cultures from IPF patients showed lower expression levels of MMP2 compared to the infected primary cultures from healthy donors with ratio of the means 0.01 (p = 0.132) (Table 3), as well as for CCL19 with ratio of the means 0.0001 (p = 0.246) and CCL21 with ratio of the means 10^−8^ (p = 0.001). However, mRNA transcript levels for MMP9 and CCR7 were higher in the cultures from IPF patients compared to the infected primary cultures from healthy donors with ratio of the means 518.41 (p = 0.324) and 21.32 (p = 0.700), respectively.

The mock infected primary cultures from IPF patients showed lower expression levels of MMP2 compared to the mock infected primary cultures from healthy donors with ratio of the means 0.01 (p = 0.051) (Table 4), as well as for CCR7 with ratio of the means 0.08 (p = 0.074), CCL19 with ratio of the means 0.0002 (p = 0.037) and CCL21 with ratio of the means 10^−8^ (p = 0.001). However, mRNA transcript levels for MMP9 were higher in the cultures from IPF patients compared to the mock infected primary cultures from healthy donors with ratio of the means 34.08 (p = 0.254).

### Innate immunity axis

**Figure 7:**
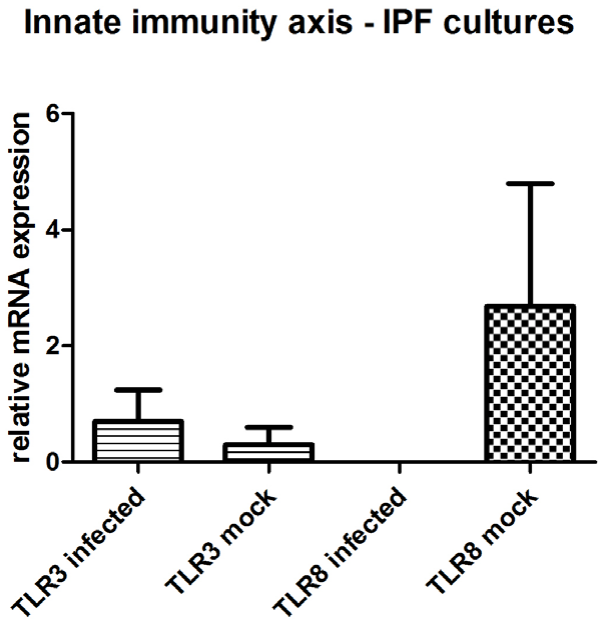
mRNA expression of innate immunity axis in HSV-1 (n = 4) and mock infected (n = 4) macrophages from IPF patients.

We investigated the expression of Toll-like receptors 3, 8 and 9 and discovered higher transcript levels of TLR3 in the infected cultures from IPF patients, while TLR8 and TLR9 were undetectable in all the culture samples from IPF patients (data not shown). Specifically, TLR3 transcript levels were upregulated in the infected cultures from IPF patients versus the mock infected cultures (mean±SD, 0.7±1.9 versus 0.3±0.6) with a ratio of 2.34. TLR8 was detected in the mock infected cultures only (mean±SD, 2.68±4.23) (Figure 7).

The transcript levels of TLR8 and TLR9 were lower in the infected cultures from healthy donors compared to the mock infected cultures (mean±SD, 0.5±0.16 versus 0.23±0.28 and 5.06±4.29 versus 49.31±24.26 respectively) with ratios of means 0.95 and 0.1 respectively. On the contrary, TLR3 transcript levels were higher in the infected cultures compared to the mock infected cultures (mean±SD, 33.33±33.33 versus 21.33±8.59) with a ratio of 1.56.

The HSV-1 infected primary cultures from IPF patients showed lower expression levels of all TLR genes compared to the infected primary cultures from healthy donors. Specifically, mRNA transcript levels for TLR3 were lower in the cultures from IPF patients compared to the infected primary cultures from healthy donors with ratio of the means 0.02 (p = 0.980). While TLR8 and TLR9 were undetectable in the cultures from IPF patients, in cultures from healthy donors the transcript levels were significant.

The mock infected primary cultures from IPF patients showed lower expression levels of TLR3 and TLR9 genes compared to the mock infected primary cultures from healthy donors. Specifically, mRNA transcript levels for TLR3 were lower in the cultures from IPF patients compared to the infected primary cultures from healthy donors with ratio of the means 0.01 (p = 0.0.28), while for TLR9 the ratio of the means was 0.00 (undetectable in the IPF cultures, p = 0.019). TLR8 transcript levels were higher in the mock infected IPF cultures compared to the cultures from healthy donors with ratio of the means 11.65 (p = 0.431).

## Discussion

The pathogenesis of IPF and other pathologic patterns of pulmonary fibrosis (non-specific interstitial pneumonia) is still obscure, as the initiating factors or injuries remain unknown. Furthermore, our understanding on the progressive and dysregulating nature of fibrosis is poor [19], [20]. Our results demonstrated the ability of Herpes Simplex Virus-type 1 to induce alterations of lung macrophages in bronchoalveolar lavage fluid that likely promote fibrotic inflammation and extracellular matrix deposition, thus, demonstrating a role for HSV-1 in lung fibrosis. First, we detected the presence of HSV-1 in patients with fibrotic idiopathic interstitial pneumonias, since the virus presented similar incidence in two different biological samples, tissue and bronchoalveolar lavage fluid. Secondly, we found that the presence of HSV-1 can enhance fibrosis by inducing the transcription of molecular pathways which promote fibrotic, angiogenetic, wound healing and innate immunity processes, suggesting a probable role of infectious factors in the pathogenesis of lung fibrosis. Furthermore, we identified infected lung macrophages as a source of profibrotic chemokines. Moreover, supporting the aforementioned findings, a different behaviour has been detected in infected macrophages of healthy controls.

The spatial and temporal variance of fibrotic lesions suggests that a repeated stimulus, such as a viral infection, causes lung injury over the course of the disease [19], [20]. Viruses are potential candidates for a role in IPF due to their ubiquitous incidence in humans and due to the nature of their lifecycle [3]. The most compelling evidence for a viral co-factor in IPF comes from studies concerning the association between the gamma-herpesvirus, EBV [3]–[9], [14], while data from human studies are limited and have produced conflicting results [5]–[9]. For instance, latent EBV infection is most often found in B cells [21]; however, in patients with IPF, EBV was found in lung tissue, including epithelial cells [22], [23]. Our results are not in disagreement with the aforementioned studies, as we did not evaluate the incidence of EBV in our samples. Regarding HHV8 incidence in IPF, previous studies are in disagreement [5], [9]. To note, that the increased frequency of HHV8 in the lungs of our IPF cohort was particularly important as HHV8 infection is predominantly found in patients with HIV infection and Kaposi's sarcoma [24], and all of the subjects tested negative for HIV in this study [5]. We also detected HHV6 in a similar frequency in both patients and controls, however, only in the BALF. Given the lack of previous data in the literature regarding this virus and the absence of any statistical difference between patients and controls, we did not further analyze this finding. It is obvious, that larger scale studies are needed in order to detect nonhuman DNA in all forms of pulmonary fibrosis [20].

Animal models support the hypothesis linking herpesvirus infections with specific T helper 2 (Th2) cytokine profiles and alternative macrophage activation [5], [15], [16] as co-factors for the development of pulmonary fibrosis [4]. Specifically, a murine gamma-herpesvirus 68 (MHV68) model of pulmonary fibrosis has enabled the identification of pathogenic cells and mediators that are believed to be important in human fibrotic disease, and they have facilitated further exploration of a pathogenic role for viruses in humans [25].

There is a limited understanding regarding the fibrotic effects of gamma-herpes viruses on cell types resident or circulating in the lung, particularly following the clearance of acute lytic infection [3], [26]. The aforementioned findings provide clues concerning the role for lytic γHV-68 infection in fibrosis, however, most humans harbor latent herpesviruses throughout their lifetime, and IPF normally occurs at an advanced age [27].

The present study explored links between IPF and herpesviruses by examining the potential of the latent infection HSV-1 to modulate cellular inflammation, fibrosis, angiogenesis and wound healing in alveolar macrophages in the lung. The virus is pleotrophic and infects multiple different cells in the lung through multiple pathways. An increase in fibrotic markers like TGF-β1 and FGF was noted, however, not statistically significant. The fact that the results of the present study regarding the measurement of different parameters in mock and infected macrophages did not reach statistical significance, should be attributed to the small sample size and to the difficulties of attaining a larger sample size given the challenge of obtaining and sustaining primary cultures. A further support of the current study is the healthy controls' behaviour, showing a different response of macrophages in viral infection. Given the fact that there have been to date no studies implicating infectious factors in primary cell cultures from IPF and NSIP patients resulting in the induction of fibrogenic pathways, these results should be scrutinized in a qualitative rather than a quantitative manner.

The mock infected macrophages from IPF patients, showed less, or at least just as, prone to angiopoietic and fibrogenic pathway promotion compared to the mock infected macrophages from healthy donors. This may lead to the hypothesis that macrophages in IPF have already established equilibrium with the surrounding fibrotic tissue, thus lacking the need to promote such pathways, or possibly that these cells have already been altered from the cytokines circulating the fibrotic lung in a way that they no longer promote fibrosis and angiopoiesis without further stimuli. This hypothesis is further supported by the behavior of IPF macrophages infected with latent HSV-1, since there was a notable 17-fold increase of SDF-1a in IPF infected cells compared to the mock infected healthy macrophages, suggesting that given an infectious stimuli the macrophages promote at least one component of the angiopoietic axis. However, the remaining genes of both axis showed the same expression pattern, suggesting more evidently the established alterations that the macrophages in IPF have sustained, thus lacking the amount of excitability promoting fibrogenesis from infection that the healthy macrophages showed. It is possible that latent herpesvirus infection may play a role in the exacerbation of IPF, suggested from the IPF macrophages results, by promoting specific gene expression but also in the initiative damage, suggested from the healthy macrophages results. Focusing on the behavior of IPF macrophages in the pathways of tissue wound healing and of innate immunity, two pathways that represent a very active role of these cells, we observed that in the mock infected cultures IPF macrophages were more prone to have less effective tissue wound healing cytokine expression compared to the healthy macrophages. However, given a stimuli such as herpesvirus infection IPF macrophages showed an increased tissue wound healing cytokine expression. However, IPF macrophages were unable to express higher amounts of innate immunity cytokines compared to the healthy macrophages, even with an infectious stimuli present, thus further supporting the hypothesis of established alterations of these cells in the fibrotic lung.

Our results suggest the virus is associated with two quite separate pathogenetic processes - in IPF and in NSIP - with a common process in both being epithelial damage. However, it is difficult to argue or imply that this virus might drive pathogenesis in two such rather disease processes. On the other hand, it has been elegantly suggested that idiopathic UIP and idiopathic NSIP, sharing a common clinical phenotype, form a spectrum of disease with a common pathogenesis [28]. It seems likely that the balance of abnormalities in each of the key wound-healing pathways may vary between individuals. This variation is likely to be responsible for the range of clinical, radiological and pathological phenotypes observed in IPF [28]. Macrophages recruited during lung inflammation can differentiate into distinct phenotypes with specialized functions in wound healing, host defence or immune regulation [14]. Exposure to Th2 cytokines promotes alternative macrophage activation, the M2 subtype, which stimulates wound healing and fibrogenesis [26]. It has been found that MMP9 levels are elevated in patients with pulmonary fibrosis and furthermore, ablation of the MMP9 gene reduces IL-13-dependent lung remodelling [29], [30]. Moreover, it has been recently demonstrated by evaluating the pulmonary response to expression of the EBV lytic transactivator protein Zta, that cells recovered by BAL of Adv-Zta-treated mice displayed greater than 10-fold elevated MMP-9 mRNA levels than that in untreated mice [31]. On the other hand, a modest increase in TGF-β levels has been detected, suggesting a mechanism for the destruction of the basement membrane and access of inflammatory cells to the alveolar space without significant increases in TGF-β activation [31]. We also found increased MMP9 levels with a parallel increase in TGF-β, in accordance with the aforementioned results, showing the effects of HSV-1 lytic infection. In contrast, we did not detect a similar increase in MMP2 levels. It is of note that TGF-β1, the prototypic growth factor, demonstrated to be elevated in fibrotic remodeling in the lungs of patients with IPF didn't change in the infected cultures compared to the mock infected cells. Moreover, it has been recently and elegantly shown that modulation of macrophage activation following TGFβ1 activation have remarkable effects on collagen deposition, fibrocyte accumulation, BAL cell infiltration and myofibroblast hyperplasia [32]. In alignment with TGF-β1 behaviour is FGF and MMP-9 expression in controls.

Furthermore, the levels of the chemokines MCP1 and CXCL12 in BAL samples of infected mice during the chronic phase of infection were detected in IFN-γ R−/− mice during the chronic phase of infection [33]. In addition, Vannella et al [17] recently found that MHV68 latently infected alveolar epithelial cells upregulated the expression of these two chemokines and induced fibrocyte accumulation in lungs of latent MHV68 infected mice. The results of the present study are in accordance with these studies from animal models, showing that after recombinant infection with HSV-1, both components of SDF-1a are elevated in patients with IPF. Notably, down-regulation of the specific receptor CXCR4 was observed in the infected samples, while expression didn't alter in healthy controls.

In the present study, increased levels of innate immunity markers such as TLRs, specifically TLR-3 and chemotactic chemokines CCR7 and its ligands, CCL19 and CCL21 were noted. A significant increase at the expression levels of CCL21 has been detected in the presence of HSV-1 infection in controls. However, its specific receptor CCR7 didn't show a similar trend, while increased levels have been detected in IPF, in agreement with current literature [34]. CCR7 expression was significantly raised in different forms of idiopathic interstitial pneumonias, suggesting that CCR7 positive cells are activated resident pulmonary fibroblasts [34]. It was recently found that TLR stimulation led to an increase in MHV68 latency, as evidenced by an increase in viral genome-positive cells 2 weeks post-in vivo stimulation by specific TLR ligands [35]. Thus, these data demonstrate that TLR stimulation can drive MHV68 reactivation from latency and suggest that periodic pathogen exposure may contribute to the homeostatic maintenance of chronic gammaherpes virus infection through stimulating virus reactivation and reseeding latency reservoirs [35], [36]. These studies indicate that viral reactivation is essential for the progression of pulmonary fibrosis; antiviral treatment can therefore prevent progression even when initiated after the establishment of fibrosis, resulting in prolonged survival.

This study does not lack limitations since, our results can not elucidate the exact role of HSV-1 and that this virus might drive disease progression. It can not be excluded that HSV-1 might have been a co-factor in the initial damage, setting off the disease progress, but not necessarily contributing to progression. Rather, a combination of injuries potentially contributes to the emergence of pulmonary fibrosis. The trigger that initiates the development of fibrosis in IPF remains unknown. However, there is increasing evidence that a wide range of potentially injurious factors may play a role in the initiation and progression of IPF. Oxidative stress related or not to smoking attitude, environmental pollutants and dusts have all been implicated as potential causes of IPF [37]. In addition, we can not exclude the possibility that this particular virus is more prone to be carried if there is pre-existing epithelial damage. Further, experimental work is certainly needed to clarify the aforementioned findings.

These results of the resent study implicate for the first time, a role for HSV-1 in fibrotic idiopathic interstitial pneumonias. In addition, infection with Herpesvirus infection with HSV-1 induced transcription of molecular pathways that promote fibrotic and angiogenetic processes, suggesting a possible role in the pathogenesis of lung fibrosis. The findings of the present study may have significant implications in developing an antiviral strategy in patients with IPF and infected with herpesvirus. However, larger studies are needed to confirm the present results.

## Materials and Methods

### Ethics Statement

The Ethics Committee of the Medical School of the University of Crete approved the protocol and all patients and control subjects provided informed consent in written form.

### Patients

Consecutive patients from the Interstitial Lung Disease Unit of the Department of Thoracic Medicine, University Hospital of Heraklion, Crete, Greece were enrolled in the study. The diagnosis was based on internationally accepted clinical and imaging criteria [1]. In eleven cases, diagnosis was made by video-assisted thoracoscopic surgery (VATS), where the histologic diagnosis of usual interstitial pneumonia (UIP) was obtained. In the remaining cases, the diagnosis was made on the basis of clinical and high-resolution computed tomography (HRCT) criteria [1], [38].

The bronchoalveolar lavage fluid (BALF) group included 20 patients with idiopathic fibrotic pneumonias (f –IIPs) (Table 5). The diagnosis was based on internationally accepted clinical and imaging criteria. The group included 13 patients with IPF and 7 patients with non-specific interstitial pneumonia (NSIP). The control group included 6 healthy volunteers who were nonsmokers and able to provide BALF samples.

**Table 5:** The clinicopathological characteristics of the subjects.

*Bronchoalveolar Lavage group*				
Characteristics	controls	IPF	f-NSIP	p value
Number	6	13	7	
Age*	56.33±5.47	70.65±6.56	61.75±14.82	p1 0.000; p2 0.008; p3 0.005
Gender (male/female)**	4/2	11/2	3/4	p1 0.012; p2 0.013; p3 0.008
Nonsmokers**	2	6	4	NS
Smokers**	4	3	2	NS
Ex-smokers**	0	4	1	NS
FEV1*	107±6.96	79.29±3.93	94±14.54	p1 0.017; p2 NS; p3 NS
FVC*	103±10.26	86.06±4.44	101.75±19.38	p1 NS; p2 NS; p3 NS
FEV1/FVC*	78.25±5	84.47±2.05	87.25±4.05	p1 NS; p2 NS; p3 NS
DLCO*	96±25	54±6.41	75±6.49	p1 NS; p2 NS; p3 0,045
KCO*	106±22	54.01±6.41	75±6.49	P1 NS; p2 NS; p3 0,045

*Cell culture group*			
Characteristics	controls	IPF	p value
Number	3	4	
Age*	70±5.64	66.75±3.9	p = 0.000
Gender (male/female)**	3/0	3/1	NS
Nonsmokers**	3	2	NS
Smokers**	0	1	NS
Ex-smokers**	0	1	NS
FEV1*	100.4±0.8	84.75±9.15	NS
FVC*	106.2±2.52	81±7.31	NS
FEV1/FVC*	94.7±2.85	83±2.79	NS
DLCO*	100±11.05	53.25±9.65	NS
KCO*	110±10.69	61.5±8.66	NS

Values are expressed as means ± SEM (standard error of the mean).

* *t*-test; *P*<0.05 is considered statistically significant.

** χ^2^ test; *P*<0.05 is considered statistically significant.

NS; not significant.

Abbreviations: FEV1: forced expiratory volume in one second, FVC: forced vital capacity, DL_CO_, diffusing capacity for carbon monoxide, KCO: DLCO per unit lung volume.

p1: *P* value between IPF and controls, p2: *P* value between f-NSIP and controls, p3: *P* value between IPF and f-NSIP.

For the primary cell cultures, bronchoalveolar lavage fluid was obtained from 4 patients with diagnosed IPF, as described above. As controls for the primary cell cultures, 3 consecutive never-smokers from those undergoing bronchoscopy for investigation of solitary nodule were recruited. These patients had negative bronchoscopy and were diagnosed as having benign nodule.

Only nonatopic subjects—atopy assessed by skin prick tests—with no history of respiratory tract infection or IPF exacerbation 12 weeks prior to bronchoscopy were studied. Microbiological evaluation of BALF was negative in all cases.

The lung specimen group included 11 patients with IPF. The control group included 4 lung tissue specimens obtained from macroscopically healthy sites of the lung, from patients ongoing lobectomy or pneumonectomy and were verified histologically.

Demographics and pulmonary function tests of patients and controls from the Bronchoalveolar Lavage group and the cell culture group are shown in Table 5.

Patients were not under steroid and/or immunosuppressive treatment.

### Biological samples and processing

BALF was obtained fresh, from stable IPF patients at room temperature. After filtering to remove debris, the BALF from each patient was divided into two samples: one for primary cell culture while the other was centrifuged at 1,500 rpm and the supernatant and pellet were stored at −80°C. Lung tissue biopsy specimens were obtained at −20°C and stored at −80°C.

### DNA extraction and PCR amplification

BALF pellet and lung tissue biopsy samples were processed as follows: Genomic DNA was extracted using the proteinase K protocol, followed by phenol extraction and ethanol precipitation according to the standard procedures.

PCR reactions for the HHV6, HHV7 and HHV8 assays were performed using Go Taq Flexi DNA polymerase (Promega, USA). The set of primers and the PCR amplification conditions for the viruses HHV6, HHV7 and HHV8 have been previously described [39]. HSV-1 and CMV were detected by PCR using commercially available kits (Maxim Biotech, USA) following the manufacturer's instructions. The primers specifically amplify target sequences of HSV-1 *DNA polymerase* gene. According to the manufacturer, there is no cross-reaction between HSV-1 and HSV-2. All specimens were examined for the presence of amplifiable DNA using a set of primers for the *b2-microglobin* gene (*β2m*). To ensure that our PCR were sensitive enough to detect relatively low levels of viral DNA, we applied a sensitivity assay as previously described [40]. The sensitivity of detection of each herpes virus, reached 200 copies/ml. PCR end products were analyzed on 1% agarose gel, stained with ethidium bromide and the bands were visualized under a UV transilluminator (260 nm). All gels were scanned (AlphaInnotech, USA). Integrated density of the bands was used as a quantitate parameter and was calculated by digital image analysis (Scion Image, USA). Normalization of each herpesvirus-positive sample was achieved by the ratio of the integration density of the viral PCR band versus the corresponding density of the *β2m* gene. All PCR products were subjected to direct sequencing, confirming the specificity of the amplification products.

### Isolation of BALF macrophages

For the isolation of BALF macrophages, fresh BALF was obtained at room temperature (R/T) and passed through a Millipore filter to isolate cells in suspension from debris and mucus. To pellet cells, samples were centrifuged at 1,500 rpm for five minutes at R/T. The supernatant was discarded and the cells were resuspended in 4 ml RPMI medium, 20% FBS, 10× penicillin/streptomycin. The cells were counted in a Neubauer haemocytometer, plated in 60 mm culture dishes and incubated at 37°C in a humidified incubator with 5% CO_2_. All samples were divided between two 60 mm culture dishes while for microscopy purposes, glass coverslips were placed in the dishes before the cells were plated. Following observation using an inverted microscope, the adhered cells (macrophages) remained in the incubator and the medium was replaced every two days.

### Infection of BALF macrophages and immunofluorescence assay

Upon confluency of the primary culture, the cells were infected with a wild-type HSV-1 strain 17+ at a multiplicity of infection (MOI) 2. Each sample was either infected with HSV-1 or mock infected, in a 60 mm culture dish. For the mock infections, the medium used for the dilution of the virus was applied to the cells, instead of the virus. The proportion of infected cells was determined by immunofluorescence, counting the number of fluorescence-positive cells in comparison to the total number of cells in each sample. Infection of the cells at a multiplicity of infection MOI = 2 resulted in 80% infectivity by HSV-1.

At specific time points corresponding to the expression of immediate-early, early and late HSV-1 genes (3 hours for ICP0, 8 hours for ICP8 and 15 hours for gG, respectively) the glass coverslips from both Petri dishes were moved to 24-well culture plates and proceeded for immunofluorescence staining, whereas the remaining cells in the dishes were recovered with Trizol reagent.

The glass coverslips were washed in PBS, fixed with 3.7% formaldehyde in PBS containing 2% sucrose) and then permeabilized with 0.5% NP-40 in PBS with 10% sucrose. The coverslips were incubated for one hour at room temperature with primary antibodies for the HSV-1 ICP0, ICP8 and gG proteins (SantaCruz, USA) diluted in PBS containing 1% fetal calf serum and subsequently washed 3 times prior to incubation with a mouse Alexa 488 secondary antibody (Molecular Probes, USA) under the same conditions. Nuclei were stained with DAPI (4,6-diamidino-2-phenylindole). The examination was performed using an epifluorescent Leica DMIRE2 microscope, equipped with a Leica DFC300 FX digital camera. Camera image acquisition was controlled by IM50 software (Leica). Single images were exported as TIFF files from the IM50 software.

### Gene expression

BALF pellets and cells from primary cultures (both mock and HSV-1 infected) were processed using the Trizol reagent (Invitrogen, Carlsbad, CA, USA) protocol for total RNA and DNA extraction according to the manufacturer's instructions.Prior of RNA extraction, a proportion of the cells in suspension was stained with Trypan Blue and then visually examined to determine the percentage of viable cells. The vast majority of the cells, approximately 95%, were viable. RNA concentration and purity were evaluated by a spectrophotometer. Aliquots of RNA were stored at −80°C until use. cDNA from each sample was derived by reverse transcription of 2 µg of total RNA using the AffinityScript™ Multi Temperature cDNA synthesis kit, (Stratagene, La Jolla, CA, USA). Random hexamers were used as amplification primers. To remove the RNA template, cDNA was incubated with *E. coli* RNaseH, and stored at −20°C until use.

The expression of cellular genes was determined 24 hours post infection, when the life cycle of HSV-1 is completed. Transcript levels of SDF1a, SDF1b, VEGF, CXCR4, TGFβ1, FGF, CCR7, CCL19, CCL21, TLR3, TLR8, TLR9, MMP2 and MMP9 were determined using the Mx3000P Real-Time PCR system (Stratagene) and SYBR Green I Master Mix (Stratagene, La Jolla, CA, USA) according to the manufacturer's instructions, as previously described [41]–[44]. All primers were designed to span at least one intron in order to avoid amplification of contaminating genomic DNA. *β-actin* was used as an internal control to normalize mRNA expression levels, as previously described [41]. To verify the results of the melt curve analysis, PCR products were analyzed by electrophoresis on 2% agarose gels stained with ethidium bromide and photographed on a UV light transilluminator. Primer sequences and annealing temperatures for all the genes analyzed, as well as for *β-actin* are shown in Table 6.

**Table 6:** Primer sequences used for quantitative Real-Time RT-PCR.

Gene	Primer pair sequence (5′-3′)	Annealing temperature
*β-Actin*	FOR: CGGCATCGTCACCAACTGREV: GGCACACGCAGCTCATTG	60°C
*TLR-3*	FOR:ACACCATCTATTAAAAGACCCATTATREV: TCCAGATTTTGTTCAATAGCTTGTT	62°C
*TLR-8*	FOR:GCTTTCTTTCTGAAGTCAGTAGTCTREV:TTTCCGTGTAGTTCCAACATAGATAA	58°C
*TLR-9*	FOR:CTGAGTGAGAACTTCCTCTACAAATGREV:TCTTTTGGTAATTGAAGGACAGGTTA	58°C
*MMP2*	FOR:CACGCTGGGCCCTCTCACTCREV:GGGCCCTCGTATACCGCATCATTC	60°C
*MMP9*	FOR:GAGTGGCAGGGGGAAGATGC REV:CCTCAGGGCACTGCAGGATG	60°C
*SDF-1a*	FOR: TGAGAGCTCGCTTTGAGTGAREV: CACCAGGACCTTCTGTGGAT	55°C
*SDF-1b*	FOR: CTAGTCAAGTGCGTCCACGAREV: GGACACACCACAGCACAAAC	55°C
*CXCR4*	FOR: GGTGGTCTATGTTGGCGTCTREV: TGGAGTGTGACAGCTTGGAG	55°C
*FGF*	FOR: CTGGCTATGAAGGAAGATGGAREV: TGCCCAGTTCGTTTCAGTG	55°C
*TGF-β1*	FOR:AAGGACCTCGGCTGGAAGTGCREV: CCGGGTTATGCTGGTTGTA	62°C
*CCL21*	FOR:CCCAGGACCCAAGGCAGTGATREV:GGGGGGCAAGAAGGATAG	58°C
*CCR7*	FOR:GGTGGTGGCTCTCCTTGTCATTTTREV:AGTTCCGCACGTCCTTCTT	55°C
*CCL19*	FOR:GGACTTCCCCAGCCCCAACTCTREV:TAACTGCTGCGGCGCTTCATCTT	62°C
*VEGF*	FOR: ATGACGAGGGCCTGGAGTGTGREV:CCCTATGTGCTGGCCTTGGTGAG	60°C

### Statistical analysis

The Kolmogorov-Smirnov test was used to determine whether the expression data obtained follow a normal distribution pattern. The mRNA expression of all genes was compared between the groups of normal and pathological samples, as well as between groups with different histological features using non-parametric procedures (the Kruskal Wallis and Mann-Whitney tests). Paired t-test (parametric analysis) was performed for the gene expression of the primary cultures.

Probability values (P-values)<0.05 were considered statistically significant. Statistical calculations were performed using SPSS 11.5 software (SPSS, Chicago, IL, USA).

## Supporting Information

Table S1 Detection of Herpesviruses DNA in BALF and lung tissue.
(DOC)

## References

[1] American Thoracic Society (2000). Idiopathic pulmonary fibrosis: diagnosis and treatment. International consensus statement. American Thoracic Society (ATS), and the European Respiratory Society (ERS).. Am J Respir Crit Care Med.

[2] Antoniou KM, Pataka A, Bouros D, Siafakas NM (2007). Pathogenetic pathways and novel pharmacotherapeutic targets in idiopathic pulmonary fibrosis.. Pulm Pharmacol Ther.

[3] Stoolman JS, Vannella KM, Coomes SM, Wilke CA, Sisson TH (2010). Latent infection by gammaherpesvirus stimulates pro-fibrotic mediator release from multiple cell types.. Am J Physiol Lung Cell Mol Physiol (Epub ahead of print).

[4] Vannella KM, Moore BB (2008). Viruses as co-factors for the initiation or exacerbation of lung fibrosis.. Fibrogenesis Tissue Repair.

[5] Tang YW, Johnson JE, Browning PJ, Cruz-Gervis RA, Davis A (2003). Herpesvirus DNA is consistently detected in lungs of patients with idiopathic pulmonary fibrosis.. J Clin Microbiol.

[6] Kelly BG, Lok SS, Hasleton PS, Egan JJ, Stewart JP (2002). A rearranged form of Epstein-Barr virus DNA is associated with idiopathic pulmonary fibrosis.. Am J Respir Crit Care Med.

[7] Stewart JP, Egan JJ, Ross AJ, Kelly BG, Lok SS (1999). The detection of Epstein-Barr virus DNA in lung tissue from patients with idiopathic pulmonary fibrosis.. Am J Respir Crit Care Med.

[8] Wangoo A, Shaw RJ, Diss TC, Farrell PJ, du Bois RM (1977). Cryptogenic fibrosing alveolitis: lack of association with Epstein-Barr virus infection.. Thorax.

[9] Zamo A, Poletti V, Reghellin D, Montagna L, Pedron S (2005). HHV-8 and EBV are not commonly found in idiopathic pulmonary fibrosis.. Sarcoidosis Vasc Diffuse Lung Dis.

[10] Efstathiou S, Ho YM, Hall S, Styles CJ, Scott SD (1990). Murine herpesvirus 68 is genetically related to the gammaherpesviruses Epstein-Barr virus and herpesvirus saimiri.. J Gen Virol.

[11] Lok SS, Haider Y, Howell D, Stewart JP, Hasleton PS (2002). Murine gammaherpes virus as a cofactor in the development of pulmonary fibrosis in bleomycin resistant mice.. Eur Respir J.

[12] Mora AL, Woods CR, Garcia A, Xu J, Rojas M (2005). Lung Infection with gamma herpesvirus induces progressive pulmonary fibrosis in Th2 biased mice.. Am J Physiol Lung Cell Mol Physiol.

[13] McMillan TR, Moore BB, Weinberg JB, Vannella KM, Fields WB (2008). Exacerbation of established pulmonary fibrosis in a murine model by gammaherpesvirus.. Am J Respir Crit Care Med.

[14] Mora AL, Torres-Gonzalez E, Rojas M, Corredor C, Ritzenthaler J (2006). Activation of alveolar macrophages via the alternative pathway in herpesvirus-induced lung fibrosis.. Am J Respir Cell Mol Biol.

[15] Mora AL, Torres-Gonzalez E, Rojas M, Xu J, Ritzenthaler J (2007). Control of virus reactivation arrests pulmonary herpesvirus-induced fibrosis in IFN-gamma receptor-deficient mice.. Am J Respir Crit Care Med.

[16] Sides MD, Klingsberg RC, Shan B, Gordon KA, Nguyen HT (2010). The Epstein-Barr virus LMP 1 and TGF-{beta}1 synergistically induce EMT in lung epithelial cells.. Am J Respir Cell Mol Biol (Epub ahead of print).

[17] Vannella KM, Luckhardt TR, Wilke CA, van Dyk LF, Toews GB (2010). Latent herpesvirus infection augments experimental pulmonary fibrosis.. Am J Respir Crit Care Med.

[18] Malizia AP, Lacey N, Walls D, Egan JJ, Doran PP (2009). CUX1/Wnt signaling regulates epithelial mesenchymal transition in EBV infected epithelial cells.. Exp Cell Res.

[19] Gross TJ, Hunninghake GW (2001). Idiopathic pulmonary fibrosis.. N Engl J Med.

[20] Center DM (2007). Taking the “idio” out of idiopathic pulmonary fibrosis: a call to arms.. Am J Respir Crit Care Med.

[21] Kutok JL, Wang F (2006). Spectrum of Epstein-Barr virus-associated diseases.. Annu Rev Pathol.

[22] Marzouk K, Corate L, Saleh S, Sharma OP (2005). Epstein-Barr-virus-induced interstitial lung disease.. Curr Opin Pulm Med.

[23] Lawson WE, Crossno PF, Polosukhin VV, Roldan J, Cheng DS (2008). Endoplasmic reticulum stress in alveolar epithelial cells is prominent in IPF: association with altered surfactant protein processing and herpesvirus infection.. Am J Physiol Lung Cell Mol Physiol.

[24] Moore PS (2000). The emergence of Kaposi's sarcoma-associated herpesvirus (human herpesvirus 8). N Engl J Med.

[25] Moore BB, Hogaboam CM (2008). Murine models of pulmonary fibrosis.. Am J Physiol Lung Cell Mol Physiol.

[26] Mosser DM, Edwards JP (2008). Exploring the full spectrum of macrophage activation.. Nat Rev Immunol.

[27] Selman M, Rojas M, Mora AL, Pardo A (2010). Aging and interstitial lung diseases: unraveling an old forgotten player in the pathogenesis of lung fibrosis.. Semin Respir Crit Care Med.

[28] Maher TM, Wells AU, Laurent GJ (2007). Idiopathic pulmonary fibrosis: multiple causes and multiple mechanisms?. Eur Respir J.

[29] Suga M, Iyonaga K, Okamoto T, Gushima Y, Miyakawa H (2000). Characteristic elevation of matrix metalloproteinase activity in idiopathic interstitial pneumonias.. Am J Respir Crit Care Med.

[30] Lanone S, Zheng T, Zhu Z, Gushima Y, Miyakawa H (2002). Overlapping and enzyme-specific contributions of matrix metalloproteinases-9 and -12 in IL-13-induced inflammation and remodeling.. J Clin Invest.

[31] Guenther JF, Cameron JE, Nguyen HT, Wang Y, Sullivan DE (2010). Modulation of lung inflammation by the Epstein-Barr virus protein Zta.. Am J Physiol Lung Cell Mol Physiol.

[32] Murray LA, Chen Q, Kramer MS, Hesson DP, Argentieri RL (2011). TGF-beta driven lung fibrosis is macrophage dependent and blocked by Serum amyloid P.. Int J Biochem Cell Biol.

[33] Krug LT, Torres-González E, Qin Q, Sorescu D, Rojas M (2010). Inhibition of NF-kappaB signaling reduces virus load and gammaherpesvirus-induced pulmonary fibrosis.. Am J Pathol.

[34] Choi ES, Pierce EM, Jakubzick C, Carpenter KJ, Kunkel SL (2006). Focal interstitial CC chemokine receptor 7 (CCR7) expression in idiopathic interstitial pneumonia.. J Clin Pathol.

[35] Gargano LM, Forrest JC, Speck SH (2009). Signaling through Toll-like receptors induces murine gammaherpesvirus 68 reactivation in vivo.. J Virol.

[36] Rudd BD, Smit JJ, Flavell RA, Alexopoulou L, Schaller MA (2006). Deletion of TLR3 alters the pulmonary immune environment and mucus production during respiratory syncytial virus infection.. J Immunol.

[37] Antoniou KM, Hansell DM, Rubens MB, Marten K, Desai SR (2008). Idiopathic pulmonary fibrosis: outcome in relation to smoking status.. Am J Respir Crit Care Med.

[38] American Thoracic Society; European Respiratory Society. (2002). American Thoracic Society/European Respiratory Society international multidisciplinary consensus classification of the idiopathic interstitial pneumonias.. Am J Respir Crit Care Med.

[39] Neofytou E, Sourvinos G, Asmarianaki M, Spandidos DA, Makrigiannakis A (2009). Prevalence of human herpes virus types 1–7 in the semen of men attending an infertility clinic and correlation with semen parameters.. Fertility and Sterility.

[40] Panagiotakis SH, Soufla G, Baritaki S, Sourvinos G, Passam A (2007). Concurrent CMV and EBV DNAemia is significantly correlated with a delay in the response to HAART in treatment-naive HIV type 1-positive patients.. AIDS Res Hum Retroviruses.

[41] Antoniou KM, Soufla G, Proklou A, Margaritopoulos G, Choulaki C (2009). Different activity of the biological axis VEGF-Flt-1 (fms-like tyrosine kinase 1) and CXC chemokines between pulmonary sarcoidosis and idiopathic pulmonary fibrosis: a bronchoalveolar lavage study.. Clin Dev Immunol.

[42] Margaritopoulos GA, Antoniou KM, Karagiannis K, Samara KD, Lasithiotaki I (2010). Investigation of Toll-like receptors in the pathogenesis of fibrotic and granulomatous disorders: a bronchoalveolar lavage study.. Fibrogenesis Tissue Repair.

[43] Economidou F, Antoniou KM, Soufla G, Lasithiotaki I, Karagiannis K (2010). Role of VEGF-stromal cell-derived factor-1alpha/CXCL12 axis in pleural effusion of lung cancer.. J Recept Signal Transduct Res.

[44] Antoniou KM, Soufla G, Lymbouridou R, Economidou F, Lasithiotaki I (2010). Expression analysis of angiogenic growth factors and biological axis CXCL12/CXCR4 axis in idiopathic pulmonary fibrosis.. Connect Tissue Res.

