# Supplementary material for: Detection of Herpes Simplex Virus Type-1 in Patients with Fibrotic Lung Diseases
Source: PLoS One. 2011 Dec 20;6(12):e27800. doi: 10.1371/journal.pone.0027800 (PMC3243679; doi:10.1371/journal.pone.0027800)
Supplement: Table S1 — Detection of Herpesviruses DNA in BALF and lung tissue. (DOC) [file pone.0027800.s001.doc]

Supplementary Table 1. Detection of Herpesviruses DNA in BALF and lung tissue.

| **Patients** | **Diagnosis** | **HSV-1**  **(norm. value)** | **CMV** | **HHV6**  **(norm. value)** | **HHV7** | **HHV8** |
| --- | --- | --- | --- | --- | --- | --- |
| Patient 1 | IPF | - | - | - | - | - |
| Patient 2 | IPF | - | - | - | - | - |
| Patient 3 | IPF | - | - | + (3.1) | - | - |
| Patient 4 | IPF | - | - | + (1.7) | - | - |
| Patient 5 | IPF | - | - | - | - | - |
| Patient 6 | IPF | - | - | + (2.1) | - | - |
| Patient 7 | IPF | - | - | - | - | - |
| Patient 8 | IPF | - | - | + (2.6) | - | - |
| Patient 9 | IPF | - | - | - | - | - |
| Patient 10 | IPF | - | - | - | - | - |
| Patient 11 | IPF | - | - | - | - | - |
| Patient 12 | IPF | - | - | - | - | - |
| Patient 13 | IPF | - | - | + (2.8) | - | - |
| Patient 14 | NSIP | - | - | + (3.2) | - | - |
| Patient 15 | NSIP | + (2.3) | - | + (1.8) | - | - |
| Patient 16 | NSIP | + (2.8) | - | - | - | - |
| Patient 17 | NSIP | - | - | + (2.4) | - | - |
| Patient 18 | NSIP | - | - | - | - | - |
| Patient 19 | NSIP | - | - | + (2.6) | - | - |
| Patient 20 | NSIP | - | - | - | - | - |
| Patient 21 | control | - | - | - | - | - |
| Patient 22 | control | - | - | + (2.1) | - | - |
| Patient 23 | control | - | - | + (2.7) | - | - |
| Patient 24 | control | - | - | + (2.9) | - | - |
| Patient 25 | control | - | - | - | - | - |
| Patient 26 | control | - | - | - | - | - |
| **Patients** | **Diagnosis** | **HSV-1**  **(norm. value)** | **CMV** | **HHV6** | **HHV7** | **HHV8** |
| Patient 1 | IPF | - | - | - | - | - |
| Patient 2 | IPF | ( 2.7) | - | - | - | - |
| Patient 3 | IPF | - | - | - | - | - |
| Patient 4 | IPF | - | - | - | - | - |
| Patient 5 | IPF | - | - | - | - | - |
| Patient 6 | IPF | - | - | - | - | - |
| Patient 7 | IPF | - | - | - | - | - |
| Patient 8 | IPF | - | - | - | - | - |
| Patient 9 | IPF | - | - | - | - | - |
| Patient 10 | IPF | - | - | - | - | - |
| Patient 11 | IPF | - | - | - | - | - |
| Patient 12 | control | - | - | - | - | - |
| Patient 13 | control | - | - | - | - | - |
| Patient 14 | control | - | - | - | - | - |
| Patient 15 | control | - | - | - | - | - |

Norm. value: normalization value
